# Evaluation of Antioxidant Activity of *Tetracarpidium conophorum* (Müll. Arg) Hutch & Dalziel Leaves

**DOI:** 10.1155/2011/976701

**Published:** 2011-09-06

**Authors:** O. U. Amaeze, G. A. Ayoola, M. O. Sofidiya, A. A. Adepoju-Bello, A. O. Adegoke, H. A. B. Coker

**Affiliations:** ^1^Department of Pharmaceutical Chemistry, Faculty of Pharmacy, University of Lagos, College of Medicine Campus, PMB 12003, Surulere, Lagos, Nigeria; ^2^Department of Pharmacognosy, Faculty of Pharmacy, University of Lagos, College of Medicine Campus, PMB 12003, Surulere, Lagos, Nigeria

## Abstract

This study evaluated the antioxidant activity as well as bioflavonoid content of the methanol and ethanol-water extracts of the fresh and dried leaves of *Tetracarpidium conophorum*. Antioxidant activity was determined by spectrophotometric methods using DPPH free radical, nitric oxide radical inhibition and ferric reducing antioxidant power assays. In addition, total phenolics, flavonoids and proanthocyanidin content were also determined. The ethanol: water extract of the dried leaves had the highest antioxidant activity with a 50% inhibition of DPPH at a concentration of 0.017 mg/mL compared to the standards, Vitamin C and Vitamin E with inhibition of 0.019 and 0.011 mg/mL, respectively. This extract also showed nitric oxide radical inhibition activity comparable to that of rutin, 54.45% and 55.03% for extract and rutin, respectively, at 0.1 mg/mL. Ferric reducing power was also comparable to that of ascorbic acid (281 and 287 **μ**M Fe (11)/g, resp.) at a concentration of 1 mg/mL. The methanol extract of both the dried and the fresh leaves had higher phenolic, flavonoids and proanthocyanidin content than the ethanol : water extract. The study reveals that *T. conophorum* can be an interesting source of antioxidants with their potential use in different fields namely food, cosmetics and pharmaceuticals.

## 1. Introduction

Significant scientific evidence has shown that, under situations of oxidative stress, reactive oxygen species (ROS) such as superoxide, hydroxyl and peroxyl radicals are generated, and the balance between antioxidation (reduction) and oxidation is believed to be a critical concept for maintaining a healthy biological system [1]. These ROS play an important role in the etiology and pathophysiology of human aging [2] and diseases such as cancer [3], coronary heart disease [4], Alzheimer's disease [5] and other neurodegenerative disorders [6], atherosclerosis [7], cataracts [8], and inflammation [9, 10]. Naturally there is a dynamic balance between the amount of free radicals produced in the body and antioxidants to scavenge or quench them to protect the body against deleterious effects. The amount of antioxidant principles present under normal physiological conditions may be insufficient to neutralize free radicals generated under pathological conditions. Therefore, it is important to enrich our diet with antioxidants to protect against harmful diseases. Hence, there has been an increased interest in the food industry and in preventive medicine in the development of “Natural antioxidants” from plant materials.

The plant *Tetracarpidium conophorum *(Mull. Arg) Hutch & Dalziel Syn. *Plukenetia conophora* commonly called African Walnut belongs to the family Euphorbiaceae. It is a climber found in the wet part of Southern Nigeria and West Africa in general. Its habitat is usually large trees; the fruits are greenish with four round seeds in each fruit. The seed testa is hard, and the cotyledons are white in colour [11]. The fruits are edible; the plant is medicinal and used for various purposes [12]. The leaves, bark, and fruit of *T. conophorum* are used medicinally, and their uses include masticatory, giddiness, thrush, antihelminthic, toothache, syphilis, dysentery, and as an antidote to snakebite [13]. In the Southern Nigeria ethnomedicine, African walnut is used as a male fertility agent and in the treatment of dysentery [14]. The methanol and ethylacetate extracts of *T. conophorum* leaves have been shown to possess good antibacterial activities especially against Gram +ve organisms [14].

The objective of this research is to determine the antioxidant activity of the fresh and dried leaves of *T. conophorum *by investigating the DPPH radical scavenging activity, ferric reducing antioxidant power assay (FRAP), and nitric oxide radical inhibition assay. 

## 2. Materials and Methods

### 2.1. Plant Materials

The leaves of *T. conophorum* used in this study were collected from a private farmland in Amichi town, Nnewi South Local Government Area of Anambra State, Nigeria, in the evening in the month of April, 2009. The leaves were authenticated by Mr. Odewo at the Forestry Research Institute of Nigeria (FRIN), Ibadan with voucher specimen number FHI 108028. Sample of the plant has been deposited in the FRIN herbarium. Some of the plant materials were dried at room temperature (25  ± 2°C) and milled into uniform dry powder. Two solvent systems were used for the extraction: methanol and a mixture of ethanol : water (70 : 30). 200 g of the fresh leaves were macerated in about 1200 mL of methanol (analar grade) and about 1200 mL of ethanol : water mixture (70 : 30), respectively, for 72 hours. 80 g of the dried leaves were extracted with 700 mL of methanol (analar grade) and 700 mL of a mixture of ethanol : water (70 : 30), respectively, for 72 hours. The extracts were filtered, respectively, first through cotton wool, then Whatman filter paper no. 42 (125 mm). The solvents were completely removed by rotary evaporator. and further removal of water was carried out by freeze drying. The dry extracts were weighed, respectively, stored in clean sample bottles, and labelled as follows: 

*Extract A*: methanol extract of the fresh leaves,

*Extract B*: ethanol : water extract of the fresh leaves,

*Extract C*: methanol extract of the dried leaves,

*Extract D*: ethanol : water extract of the dried leaves.

### 2.2. Chemicals

DPPH (1,1-diphenyl-2-picryl hydrazyl), ascorbic acid, Vitamin E, rutin, catechin, gallic acid, folin Ciocalteau's reagent, and TPTZ (2,4,6-tripyridyl-s-triazine) were obtained from Sigma Aldrich chemical company, USA. All other reagents and chemicals were of analytical grade and obtained locally from BDH, Fluka and Aldrich in Nigeria.

### 2.3. Phytochemical Studies

The chemical constituents in the leaves of *T. conophorum* were classified qualitatively using phytochemical reagents according to procedures described by Trease and Evans, 1983 [15]. The leaves were screened for the presence of saponins, flavonoids, tannins, alkaloids, glycosides, and other constituents.

### 2.4. Determination of Antioxidant Activity

#### 2.4.1. Rapid Screening for Free Radical Scavenging Activity

Rapid thin layer chromatography screening for antioxidant activity was carried out by spotting a concentrated methanolic solution of each extract on silica gel plates. The plates were sprayed with 0.2% w/v DPPH in methanol. The plants were visualized for the presence of yellowish spots.

#### 2.4.2. DPPH's Radical Scavenging Activity

The radical scavenging activity of the plant extracts against 1,1-diphenyl-1-picryl-hydrazyl (Sigma-Aldrich) radical was determined by measuring UV absorbance at 517 nm. Radical scavenging activity was measured by a slightly modified method of Brand-Williams et al. [16]. The following concentrations of extracts were prepared: 0.02, 0.04, 0.06, 0.08, and 0.1 mg/mL. Ascorbic acid and *α*-tocopherol were used as standards, and the same concentrations were prepared as the test solutions. All of the solutions were prepared with methanol. Two mL of each prepared concentrations were placed into test tubes, and 0.5 mL of 1 mM DPPH solution in methanol was added thereafter. The experiments were carried out in triplicates. The test tubes were incubated for 15 min at room temperature, and the absorbance was read at 517 nm. A blank solution was prepared and measured containing the same amount of methanol and DPPH. Lower absorbance of the reaction mixture indicates higher free radical scavenging activity. The radical scavenging activity was calculated using the following formula:

(1) $$\text{DPPH}\;\text{scavenging}\;\text{effect}\;\left(\text{\%}\right)=\left[\frac{\text{AB}-\text{AA}}{\text{AB}}\right]\times 100,$$

where AB is the absorption of blank sample and AA is the absorption of tested extract solution.

#### 2.4.3. Total Antioxidant Activity (FRAP Assay)

A modified method of Benzie and Strain [17] was adopted for the ferric reducing antioxidant power (FRAP) assay. It depends on the ability of the sample to reduce the ferric tripyridyltriazine (Fe(III)-TPTZ) complex to ferrous tripyridyltriazine (Fe(II)-TPTZ) at low pH. Fe(II)-TPTZ has an intensive blue colour which can be read at 593 nm. 1.5 mL of freshly prepared FRAP solution, containing 25 mL of 300 mM acetate buffer pH 3.6, 2.5 mL of 10 mM 2,4,6-tripyridyl-s-triazine (TPTZ) in 40 mM HCl, and 2.5 mL of 20 mM ferric chloride {FeCl_3_·6H_2_O} solution, was mixed with 1 mL of the extracts, and the absorbance was read at 593 nm. The standard curve was linear between 100 and 500 *μ*M FeSO_4_·7H_2_O. Results are expressed in *μ*M Fe(II)/g dry plant material and compared with that of ascorbic acid.

#### 2.4.4. Nitric Oxide Radical Inhibition Assay

Sodium nitroprusside in aqueous solution at physiological pH spontaneously generates nitric oxide, which interacts with oxygen to produce nitrite ions, which can be estimated by the use of the Griess Illosvoy reagent [18]. In the present investigation, the Griess Illosvoy reagent is modified by using naphthylethylenediamine dihydrochloride (0.1% w/v) instead of 1-naphthylamine (5%). The reaction mixture (3 mL) containing sodium nitroprusside (10 mM, 2 mL), phosphate buffer saline (0.5 mL), and extract or standard solution (20–100 *μ*g, 0.5 mL), was incubated at 25°C for 150 min. After incubation, 0.5 mL of the reaction mixture containing nitrite was pipetted and mixed with 1 mL of sulphanilic acid reagent (0.33% in 20% glacial acetic acid) and allowed to stand for 5 min for complete diazotization. Then 1 mL of naphthylethylenediamine hydrochloride (0.1%) was added, mixed, and allowed to stand for 30 min. A pink-coloured chromophore was formed in diffused light. The absorbance of these solutions was measured at 540 nm against the corresponding blank solutions. Rutin was used as standard.

### 2.5. Total Phenolic Content

Total phenolic content was determined according to the Folin and Ciocalteau's method, and gallic acid was used as a standard [19]. Concentrations of 0.01, 0.02, 0.03, 0.04, and 0.05 mg/mL of gallic acid were prepared in methanol. Concentrations of 0.1 and 1 mg/mL of plant extract were also prepared in methanol. 0.5 mL of each sample was mixed with 2.5 mL of a tenfold diluted Folin-Ciocalteau's reagent and 2 mL of 7.5% sodium carbonate. The mixture was allowed to stand for 30 min at room temperature before the absorbance was read at 760 nm spectrophotometrically. All determinations were performed in triplicates. The total phenolic content was expressed as gallic acid equivalent (GAE) using the following equation based on calibration curve: *y* = 11.46*x* − 0.015, *R* ^2^ = 0.987.

### 2.6. Total Flavonoid Content

Total flavonoid content was determined using a method of Miliauskas et al. [20]. To 2 mL sample was added 2 mL of 2% AlCl_3_ in ethanol. The absorbance was measured at 420 nm after 1 hr at room temperature. Concentrations of 0.1 mg/mL and 1 mg/mL of the extract in methanol were used, while rutin concentrations of 0.01, 0.02, 0.04, 0.08, and 0.10 mg/mL were used to obtain the calibration curve. Solutions were prepared in methanol. Total flavonoid content was calculated as rutin equivalent (RE) in mg/g using the following equation based on the calibration curve: *y* = 12.69*x* − 0.002, *R* ^2^ = 0.999.

### 2.7. Proanthocyanidin Content

Determination of proanthocyanidin was based on the procedure reported by Sun et al. [21]. A volume of 0.5 ml of 1.0 mg/mL of extract solution was mixed with 1.5 mL of 4% vanillin-methanol solution and 0.75 mL concentrated hydrochloric acid. The mixture was allowed to stand for 15 min after which the absorbance was measured at 500 nm. Extract samples were evaluated at a final concentration of 1 mg/mL. Total proanthocyanidin contents were expressed as catechin equivalents (mg/g) using the following equation based on the calibration curve: *y* = 3.922*x* + 0.058, *R* ^2^ = 0.992.

### 2.8. Statistical Analysis

All data were analysed using one-way ANOVA, and results presented as mean ± SEM. Graphical representations were done using bar chart or line graph. Level of significance was placed at *P* value  ≤ 0.05.

## 3. Results

### 3.1. Phytochemical Composition

Phytochemical screening of the leaves indicated the presence of tannins, phlobatannins, flavonoids, phenolic compounds, and alkaloids. However, no glycosides nor saponins were detected (Table 1).

### 3.2. DPPH Free Radical Assay

**Figure 1:**
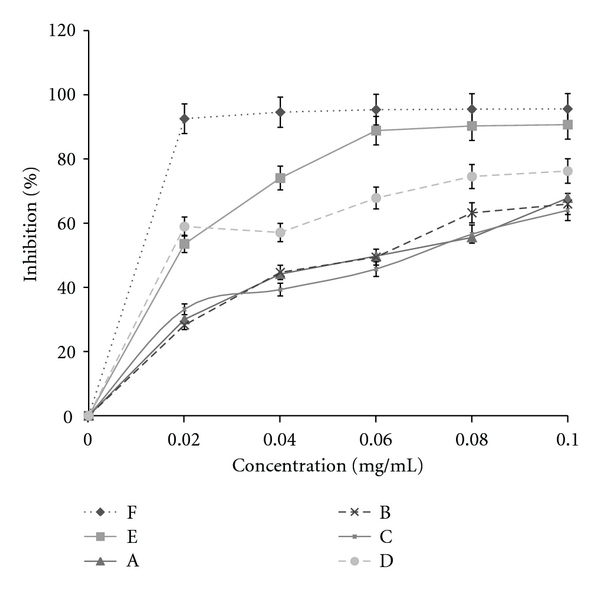
Graph comparing the DPPH's radical scavenging activity of different concentrations of Vitamins C, E, and extracts from the leaves of *T. conophorum*. Values are expressed as mean ± SEM, *n* = 3/group. A: Methanol extract of the fresh leaves. B: Ethanol : water extract of the fresh leaves. C: Methanol extract of the dried leaves. D: Ethanol : water extract of the dried leaves. E: Vitamin E. F: Vitamin C.

Rapid TLC screening for antioxidant activity was positive for the extracts where the colour of the DPPH spray changed from purple to yellowish spots. IC_50_ for DPPH inhibition was 11, 19, 62, 69, and 17 *μ*g/mL for ascorbic acid, Vitamin E, Extracts A, B, C, and D, respectively (Figure 1). Concentration at max. inhibition was also 0.02 mg/mL for vitamin C, 0.08 mg/mL for vitamin E, and 0.1 mg/mL for Extracts A, B, C, and D, respectively.

### 3.3. Total Antioxidant Activity (FRAP)

The total antioxidant activity of the extracts was 175, 200.25, 204.4, and 281 *μ*M Fe(II)/g for Extracts A, B, C, and D, respectively (Table 2). The positive control ascorbic acid had a value of 287 *μ*M Fe(II)/g.

### 3.4. Nitric Oxide Radical Inhibition

**Figure 2:**
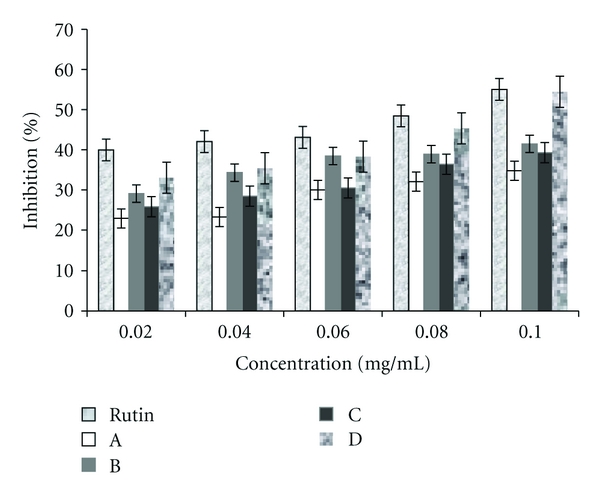
Graph comparing the nitric oxide radical inhibition activity of rutin and leaf extracts of *T. conophorum*. Values are expressed as mean ± SEM, *n* = /group3.

As illustrated in Figure 2, the plant extracts caused a low-dose-dependent inhibition of nitric oxide. At the maximum concentration (0.1 mg/mL) used, the nitric oxide radical percentage inhibition was as follows: 34.76%, 41.47%, 39.27%, 54.45%, and 55.03% for Extracts A, B, C, D, and standard rutin.

### 3.5. Total Phenolic Content

Total phenolic content of the different extracts of *T. conophorum *leaves was obtained from the regression equation of the calibration curve of gallic acid (Table 3) and expressed as gallic acid equivalent (GAE). The phenolic content of the leaves extracts are as follows: 6.16, 3. 57, 14.48, and 10.10 mg/g of plant material for Extracts A, B, C, and D, respectively. Correlation of *R* ^2^ = 0.969, 0.948, 0.988, and 0.884 for Extracts A, B, C, and D, respectively, were obtained between the data for phenolic content and DPPH inhibition.

### 3.6. Total Flavonoid Content

Total flavonoid content was obtained from the regression equation of the calibration curve of rutin (Table 3), and expressed as rutin equivalent. Total flavonoid content of the extracts are Extract A: 6.4, B: 2.51, C: 11.52, and D: 5.92 mg/g. Correlation of *R* ^2^ = 0.969, 0.948, 0.988, and 0.884 for Extracts A, B, C, and D, respectively, were obtained between the data for total flavonoid content and DPPH inhibition.

### 3.7. Proanthocyanidin Content

Proanthocyanidin was determined from the regression equation of the calibration curve of catechin (Table 3) and expressed as catechin equivalent (CE). The proanthocyanidin content of the extracts are: A: 4.8, B: 0.61, C: 29.39, and D: 2.68 mg/g. Correlation of *R* ^2^ = 0.969, 0.948, 0.988, and 0.884 for Extracts A, B, C, and D, respectively, were obtained between the data for proanthocyanidin content and DPPH inhibition.

## 4. Discussion

The DPPH test showed the ability of the test compound to act as a free radical scavenger. DPPH assay method is based on the reduction of methanolic solution of the coloured free radical, DPPH, by a free-radical scavenger. DPPH, a protonated radical, has characteristic absorbance maxima at 517 nm, which decreases with the scavenging of the proton radical. This property has been widely used to evaluate the free radical scavenging effect of natural antioxidants [22]. The stable free radical DPPH has been widely used to test the free radical-scavenging ability of various dietary antioxidants [16]. Because of its odd electron, DPPH gives a strong absorption band at 517 nm in visible spectroscopy. As this electron becomes paired off in the presence of a free radical scavenger, the absorption vanishes, and the resulting decolourization is stoichiometric with respect to the number of electrons taken up. The leaf extracts showed considerable radical scavenging activity in a concentration-dependent manner. The ethanol/water extract of the dried leaves (Extract D) exhibited a good potential to act as a free radical scavenger with IC_50_ for DPPH inhibition (0.017 mg/mL) comparable to that of Vitamin E (0.019 mg/mL) and Vitamin C (0.011 mg/mL) which are known free radical scavengers. In fact, it appeared to be slightly better than Vitamin E at 50% inhibition and about 1.5 times less potent than Vitamin C. However, a maximum inhibition was achieved at a higher concentration of 0.1 mg/mL compared to 0.02 mg/mL and 0.08 mg/mL for Vitamins C and E, respectively. Hence, a higher concentration would be required to achieve maximal inhibition of DPPH compared to Vitamins C and E. Extracts A, B, and C were about three and six times less potent than Vitamins E and C, respectively. The scavenging effect of the leaves extracts and standards on the DPPH radical decreased in the following order: ascorbic acid > *α*-tocopherol > Extract D > Extract A > Extract B > Extract C.

FRAP (Ferric reducing antioxidant power) is one of the most rapid test and very useful for routine analysis. The antioxidative activity is estimated by measuring the increase in absorbance caused by the formation of ferrous ions from FRAP reagent containing TPTZ (2,4,6-tri-(2-pyridyl)-s-triazine) and FeCl_3_·6H_2_O. It depends on the ability of the sample to reduce the ferric tripyridyltriazine (Fe(III) TPTZ) complex to ferrous tripyridyltriazine (Fe(II) TPTZ) at a low pH. (Fe(II) TPTZ) has an intensive blue colour which can be read at 593 nm [12]. The reducing ability of the extracts was in the range of 175–281 *μ*M Fe(II)/g. The ethanol : water extract of the dried leaves had a comparable reducing power as the standard ascorbic acid used. The reducing ability of the leaves extracts are in the order: Extract D > C > B > A.

 It is well known that nitric oxide has an important role in various inflammatory processes [23]. Sustained levels of production of this radical are directly toxic to tissues and contribute to the vascular collapse associated with septic shock [23]. Furthermore, chronic exposure to nitric oxide radical is associated with various carcinomas and inflammatory conditions including juvenile diabetes, multiple sclerosis, arthritis, and ulcerative colitis. The toxicity of NO increases greatly when it reacts with superoxide radical, forming the highly reactive peroxynitrite anion (ONOO) [24]. Nitric oxide is classified as a free radical because of its unpaired electron and displays important reactivity with certain types of proteins and other free radicals. 

*In vitro* inhibition of nitric oxide radical is also a measure of antioxidant activity. This method is based on the inhibition of nitric oxide radical generated from sodium nitroprusside in buffer saline and measured by the Griess reagent. The nitric oxide generated from sodium nitroprusside reacts with oxygen to form nitrite. The extract inhibits nitrite formation by directly competing with oxygen in the reaction with nitric oxide. In presence of scavengers, the absorbance of the chromophore is evaluated at 546 nm. The activity is expressed as % reduction of nitric oxide [25]. The present study showed that Extract D has nitric oxide scavenging activity comparable to that of the standard rutin. Nitric oxide scavenging activity of the extracts is in the order: Extract D> B> C> A. Extract B (ethanol : water extract of the fresh leaves) had a higher nitric oxide scavenging activity compound to Extract A which is the methanol extract of the fresh leaves. Extract D which is the ethanol : water of the dry leaves also had a higher nitric oxide scavenging activity compared to Extract C which is the methanol extract of the dry leaves. It thus appears that ethanol : water (70%) is a better solvent for extracting antioxidant molecules with NO scavenging activity. Ethanol and water mixture provides a better range of polarity for the extraction of antioxidant molecules, as ethanol will extract the less polar compounds whereas more polar compounds can also be extracted by water. Methanol will extract mainly polar molecules, hence, results also show that Extract A had a significantly higher content of phenolic compounds compared to Extract B and Extract C has a significantly higher content of phenolic compounds compared to Extract D (Table 3). It would appear that other less polar antioxidant molecules such as xanthones, carotenoids, and chlorophyll may be present which are enhancing the antioxidant effect of the ethanol : water extracts.

Plant phenolics are a major group of compounds acting as primary antioxidants or free radical scavengers [26]. It was thus reasonable to determine the phenolic content in the plant extract. The total phenolic content in the leaves extract was recorded as 6.16, 3.57, 14.48, and 10.10 mg/g plant material for Extracts A, B, C, and D, respectively. Correlation of *R* ^2^ = 0.969, 0.948, 0.988 and 0.884 for Extracts A, B, C, and D, respectively, was obtained between the data for phenolic content and DPPH inhibition. 

Flavonoids are the most common group of polyphenolic compounds in the human diet and are found ubiquitously in plants. Therefore, it was reasonable to determine the total flavonoids content in *T. conophorum* leaves extracts. The flavonoid content in the leaves extract was obtained as 6.4, 2.51, 11.52, and 5.92 mg/g plant material for Extracts A, B, C and D, respectively. There was also a good correlation between the data for flavonoid content and DPPH inhibition *R* ^2^ = 0.969, 0.948, 0.988, and 0.884 for Extracts A, B, C, and D, respectively.

Proanthocyanidins are a type of bioflavonoids that have been shown to have very potent antioxidant activity. Proanthocyanidin content in the leaves extract was obtained as 4.8, 0.61, 29.39, and 2.68 mg/g plant material for Extracts A, B, C, and D, respectively. Correlation between the proanthocyanidin content and DPPH assay were 0.969, 0.948, 0.988 and 0.884 for Extracts A, B, C, and D, respectively.

For extracts A, B, and C, it is likely that the bioflavonoids are involved or contributory to their free radical scavenging activity as indicated by the correlation between the bioflavonoids data and DPPH inhibition. Extract D had a correlation of 0.884, suggesting that the bioflavonoids are responsible for 88% of the free radical scavenging activity of this extract. However, this extract had the highest free radical scavenging activity as well as antioxidant activity. This leads to the fact there may be other bioactive compounds other than bioflavonoids present in the extract which are responsible for the observed antioxidant activity. Other non-phenolic antioxidant molecules that may be present include carotenoids [27], xanthones [28], and chlorophyll [29].

 The methanol extract of both the dried and fresh leaves had a higher phenolic, flavonoids, and proanthocyanidin content. This shows that methanol extracted the bioflavonoid contents of the leaves better than the ethanol/water mixture; however, the methanol extract of the dried leaves had higher amounts of bioflavonoids than that of the fresh leaves extracts. This suggests that some phytochemical changes may have taken place during the drying process resulting in higher amounts of these bioactive compounds. It is well known that there are variations in the type and amount of phytochemicals present in the fresh and dried leaves of a plant; for example, in the preparation of certain traditional remedies requiring pawpaw leaves, *Carica papaya* L. (which are used in many decoctions for bathing children), it is specified that the dead leaves which have actually fallen off the tree should be used rather than the green leaves. The dead leaves are usually brown and richer in phenolic constituents than the green leaves [30]. 

Many researchers have reported positive correlation between free radical scavenging activity and total phenolic compound. Oki et al. [31] observed that the radical scavenging activity increased with the increase of phenolic compound content. Flavonoids have been shown to have antibacterial, antineoplastic, antiviral, anti-inflammatory, antiallergic, antithrombotic, and vasodilatory activities [32]. The potent antioxidant activities of flavonoids have been suggested to be responsible for many of the above actions as oxidative damage is implicated in most disease processes. *T. conophorum* leaf extract can hence be exploited in the treatment of the various conditions mentioned above. Traditional use for dysentery and toothache suggest possible antibacterial and anti-inflammatory properties, respectively.

## 5. Conclusion

The extracts of *T. conophorum* leaves showed good free radical scavenging activity. The ethanol : water extract of the dried leaves had the best antioxidant activity; the broad range of antioxidant activity of this extract indicates the potential of the plant as a source of natural antioxidants or nutraceuticals with potential application to reduce oxidative stress and consequent health benefits. The methanol extract of the dried leaves also had the highest amounts of polyphenols. It can thus be concluded that the dried leaves of *T. conophorum *possess more antioxidant activity than the fresh leaves. Though the ethanol/water extract of the dried leaves showed the highest antioxidant activity, the methanol extract possesses higher amounts of plant bioflavonoids which are also known to be responsible for the antioxidant activity of most plants. The antioxidant potential of the extract D was comparable to that of Vitamin E, and about 1.5 times less potent than Vitamin C. Phenolic compounds found in plants were likely to be the main contributors of antioxidant activity of the leaves extracts, though it appears that antioxidant molecules such as xanthones, carotenoids, and chlorophyll may be contributing to the antioxidant properties of the ethanol : water extracts.

The plant may thus be exploited in the pharmaceutical and food industries.

## Figures and Tables

**Table 1:** Phytochemical composition of *T. conophorum* leaves.

Phytochemical substance	Result
Alkaloids	+
Flavonoids	+
Glycosides	NP
Phenolic compounds	+
Phlobatanins	+
Saponins	NP
Tannins	+

+: present; NP: not present.

**Table 2:** Total antioxidant activity (FRAP assay).

Standard/extract	FRAP^a^
A	175 ± 0.01^b^
B	200.25 ± 0.02^b^
C	204.5 ± 0.02^b^
D	281 ± 0.03^b^
Ascorbic acid	287 ± 0.07

^a^expressed in units of *μ*mol Fe(II)/g. Values are expressed as mean ± SEM, *n* = 3/group, ^b^ *P* ≤ 0.05 compared to ascorbic acid.

**Table 3:** Total phenolic, flavonoids, and proanthocyanidin content.

Extract	Total phenols^b^	Total flavonoids^c^	Proanthocyanidin content^d^
A	6.16 ± 0.03	6.4 ± 0.02	4.8 ± 0.09
B	3.57 ± 0.01	2.52 ± 0.01	0.61 ± 0.00
C	14.48 ± 0.05	11.52 ± 0.00	29.39 ± 0.09
D	10.10 ± 0.05	5.92 ± 0.01	2.68 ± 0.01

^b^expressed as mg gallic acid/g plant material.

^c^expressed as mg rutin/g plant material.

^d^expressed as mg catechin/g plant material.

Values are expressed as mean ± SEM, *n* = 3/group.
